# Agro-industrial by-product in photoheterotrophic and mixotrophic culture of *Tetradesmus obliquus*: Production of ω3 and ω6 essential fatty acids with biotechnological importance

**DOI:** 10.1038/s41598-020-63184-4

**Published:** 2020-04-14

**Authors:** Agata Piasecka, Agnieszka Nawrocka, Dariusz Wiącek, Izabela Krzemińska

**Affiliations:** Department of Physical Properties of Plant Materials, Institute of Agrophysics, Polish Academy of Sciences, Doświadczalna 4, 20-290 Lublin, Poland

**Keywords:** Biochemistry, Biotechnology

## Abstract

In recent years, researchers have highlighted the role of low cost-efficient agro-industrial by-products used as supplements in algal culture media. The aim of the study was to identify and characterize the basic metabolic pathways in *Tetradesmus obliquus* cells induced by supplementation with beet molasses in photoheterotrophic and mixotrophic culture conditions. To assess the impact of the nutritional strategy in unicellular algae, growth curves were plotted and lipid, carbohydrate, and protein levels were determined. Fourier Transform Infrared Spectroscopy was applied to measure the *Tetradesmus obliquus* cell composition. Additionally, the C16-C18 fatty acid profile of *Tetradesmus obliquus* was determined by gas chromatograph/mass spectrometry. The switch from autotrophy to photoheterotrophy and mixotrophy contributes to shortening of the adaptation growth phase. The highest protein content was obtained in the mixotrophic growth. This study has demonstrated high content of 18:1, cisΔ_9_, 18:2, cisΔ_9,12_, ω6, and 18:3, cisΔ_9,12,15_, ω3 in photoheterotrophic and mixotrophic culture conditions. High levels of proteins and essential fatty acids make *Tetradesmus obliquus* cell biomass important for human and animals health.

## Introduction

Microalgae are not only a source of many valuable bio-products e.g. proteins, carbohydrates, lipids, ω3 and ω6 fatty acids or pigments applied in many different commercial sectors but can be a simple and economical solution to wastewater treatment and waste management^1^. Algal cells utilize effectively agricultural, industrial and municipal wastewaters, waste raw materials, and by-products from many branches of industry. Algae are capable of assimilating organic compounds and other ingredients for biomass production and synthesis of both basic and specific metabolites. Additionally, the use of wastewaters and waste materials make microalgal biomass and bio-product production environmentally friendly^2^. A number of studies have described supplementation of the algal culture medium with agricultural waste such as dairy manure^3^, liquid waste produced in piggeries^4^, post-fermentation effluents and wastewater from fruit, vegetable or cultivated plant processing, i.e. residues of cane bagasse and pineapple peel^5^, cassava^6^, sweet sorghum^7^, or hydrolysate of Jerusalem artichoke tubers^8^.

Most algae are autotrophs; however, some species such as *Auxenochlorella protothecoides*^9^ *Parachlorella kessleri*^10^ and *Tetradesmus obliquus* can assimilate organic compounds. Cultivation modes based on organic carbon sources include heterotrophy, photoheterotrophy, and mixotrophy^11^. Compared to the heterotrophic mode, the photoheterotrophic and mixotrophic cultivation systems have several advantages e.g. intensification of algal growth and synthesis of valuable metabolites, such as fatty acids^12^. Green algae growing in photoheterotrophic and mixotrophic culture conditions are natural producers of polyunsaturated fatty acids^10,13^. In terms of human nutrition, one of the most important PUFAs are essential fatty acids including short chain polyunsaturated fatty acids: ω3 (α-linolenic acid; 18:3, cisΔ_9,12,15_ (ALA)) and ω6 (linoleic acid; 18:2, cisΔ_9,12_ (LA)), which are precursors for formation of long chain polyunsaturated fatty acids but cannot be synthesized by humans^14–16^. Besides application in human and animal nutrition, fatty acids are used in production of biofuels^17^. The similarity of the photoheterotrophic cultivation system to mixotrophy consists in microalgal requirements for light and the utilization of organic compounds as a carbon source. In contrast to mixotrophy, microalgae cultivated in photoheterotrophic culture conditions cannot absorb carbon dioxide^11^. Photoheterotrophy is very rarely applied in comparison with mixotrophic cultivation.

*Tetradesmus obliquus (Scenedesmus obliquus)* is considered as a suitable candidate for utilization of organic carbon sources of agricultural origin in mixotrophic culture conditions, especially in biodiesel production^17^. In turn, the photoheterotrophic cultivation of *T. obliquus* is a yet unknown area. To the best of our knowledge, this is the first study to examine the potential of beet molasses application in the production of ω3 and ω6 essential fatty acids.

**Table 1:** The influence of culture conditions on basis growth parameters, biochemical composition and fatty acid profile of *Tetradesmus obliquus*.

	*Tetradesmus obliquus*		
	autotrophy	photoheterotrophy	mixotrophy
	Growth parameters*		
Biomass yield (g L^−1^)	2.12 ± 0.15^a^	2.86 ± 0.56^a^	4.21 ± 0.74^b^
Daily biomass productivity (g L^−1^ day^−1^)	0.18 ± 0.01^a^	0.23 ± 0.04^a^	0.39 ± 0.01^b^
Specific growth rate, 0–3 (day^−1^)	0.33 ± 0.06^a^	0.67 ± 0.06^b^	0.77 ± 0.03^c^
Biomass doubling time, 0–72 (h)	51.3 ± 9.44^a^	24.9 ± 2.22^b^	21.5 ± 0.95^b^
Biochemical composition*			
Protein content (%,w/w)	24.13 ± 5.02^b^	35.36 ± 7.05^bc^	45.15 ± 7.68^c^
Protein productivity (mg L^−1^)	512.51 ± 36.74^a^	972.52 ± 175.78^b^	1902.14 ± 335.16^c^
Daily protein productivity (mg L^−1^ day^−1^)	42.71 ± 3.06^a^	81.04 ± 14.65^b^	158.51 ± 27.93^c^
Lipid content (%,w/w)	14.13 ± 5.02^a^	13.74 ± 2.09^a^	13.48 ± 2.95^a^
Lipid productivity (mg L^−1^)	299.48 ± 21.47^a^	376.80 ± 68.11^a^	568.75 ± 100.22^b^
Daily lipid productivity (mg L^−1^ day^−1^)	24.96 ± 1.79^a^	31.40 ± 5.67^a^	47.39 ± 8.35^b^
Carbohydrates content (%,w/w)	61.82 ± 0.98^a^	32.62 ± 3.54^b^	24.97 ± 3.20^c^
Carbohydrates productivity (mg L^−1^)	1295.63 ± 92.89^a^	896.61 ± 162.06^b^	1053.23 ± 185.58^c^
Daily carbohydrates productivity (mg L^−1^ day^-1^)	107.97 ± 77.74^a^	87.77 ± 15.46^b^	74.71 ± 13.50^b^
Fatty acid profile*			
Total C16–18 (%,w/w)	79.99 ± 2.27^a^	78.73 ± 5.32^a^	78.12 ± 5.96^a^
SFA^•^	33.07 ± 2.03^a^	21.21 ± 2.05^b^	20.34 ± 3.05^b^
MUFA^••^	35.76 ± 2.03^a^	26.76 ± 1.93^b^	23.22 ± 2.80^c^
PUFA^•••^	11.16 ± 3.58^a^	30.77 ± 4.10^b^	34.56 ± 5.29^b^
ω6:ω3 ratio	3.98 ± 0.73^a^	1.58 ± 0.35^b^	1.45 ± 0.37^b^

*the results are presented as the means of 9 measurements from three biological replicates ±SD, means followed by the same letter are not significantly different; Tukey HSD test, α = 0.05.

^•^-saturated fatty acids.

^••^- monounsaturated fatty acids.

^•••^- polyunsaturated fatty acids.

The aim of this study is to identify and characterize the basic metabolic pathways in unicellular green algae *T. obliquus* induced by supplementation with beet molasses as a source of carbon and nitrogen. The study investigates the effect of autotrophic, photoheterotrophic, and mixotrophic culture conditions on growth parameters and biochemical composition with special consideration of the C16–18 fatty acid profile in *T. obliquus* cells.

## Results

### Growth characteristics of *T. obliquus*

**Figure 1:**
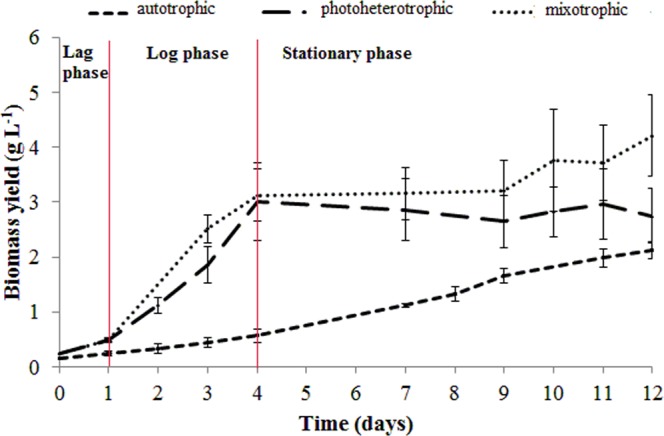
Growth curve of *Tetradesmus obliquus* under autotrophic, photoheterotrophic, and mixotrophic culture conditions with marked growth phases; lag, log, and stationary (the results are presented as the means of 9 measurements from three biological replicates, error bars represent SD).

The *T. obliquus* growth depending on the culture conditions is presented in Fig. 1. Three culture variants were used, i.e. autotrophic, photoheterotrophic, and mixotrophic, which corresponded to the aerated culture (control), molasses-supplemented culture, and aerated molasses-supplemented culture. The algal growth on the liquid medium was illustrated graphically as growth curves. The analysis of the curves indicated the individual growth phases. The course of the growth curves depends on the conditions used in the experiment. A linear phase was observed in the autotrophic culture. In the photoheterotrophic culture, there was a short lag phase (0–1 day) followed by a log phase (1–4 days) and a stationary phase (4–12 days). As in the photoheterotropic culture, the mixotrophic variant was characterised by a short lag phase (0–1 day) and a log phase (1–4 days). The mixotrophic culture growth declined, but no typical stationary phase was noted.

The growth conditions exert a significant effect on the biomass yield, daily biomass productivity, and growth parameters in *T. obliquus*, i.e. the specific growth rate (0–3 days) and the biomass doubling time (0–72 h). Table 1 presents all parameters of *T. obliquus* growth. In the autotrophic variant, 2.12 g L^−1^ of *T. obliquus* cell biomass were obtained after 12 days of cultivation. The highest increase in *T. obliquus* biomass, i.e. 4.21 g L^−1^, was noted in the mixotrophic culture. Simultaneously, the level of daily biomass productivity was estimated at 0.39 g L^−1^ day^−1^. The specific growth rate in *T. obliquus* increased in the following order: autotrophic<photoheterotrophic<mixotrophic. The *T. obliquus* biomass doubling time in the control culture was over 51 h and 2.4-fold shorter in the mixotrophic culture.

**Table 2:** Location of maxima of FTIR absorption bands for *T. obliquus* cell biomass.

Species	Nutritional strategy			Type of binding and functional group	Macromolecule	Wavenumber range (cm^−1^)^40,56,57^
*Tetradesmus obliquus*	autotrophy	photoheterotrophy	mixotrophy			
Wavelenghts (cm^-1^)	3286	3288	3288	νO-H/νN-H^•^	H_2_O/proteins	3400–3200
	2954	2956	2956	ν_as_CH_3_	lipids	2960
	2917	2917	2917	ν_as_CH_2_		2930
	2850	2850	2850	νCH_2_, νCH_3_		2850
	1731	1735	1739	νC=O		1740
	1643	1643	1643	νC=O	proteins	1650
	1536	1535	1536	δN-H/νC-N		1540
	1454	1450	1454	δ_as_CH_2_, δ_as_CH_3_		1455
	1376	1378	1382	δ_as_CH_2_, CH_3_/δC-O		1390
	1238	1238	1238	ν_as_P=O	nukleic acids	1240
	1149	1151	1151	νC-O-C	carbohydrates	1200–900
	1072	1072	1072	νC-O-C/ νP=O		
	1020	1025	1029	νC-O-C		

^•^ν = symmetric stretching, ν_as_ = asymmetrical stretching, δ = symmetric deformation, δ_as_ = asymmetrical deformation.

### Analysis of *T. obliquus* biomass by Fourier Transform Infrared Spectroscopy

**Figure 2:**
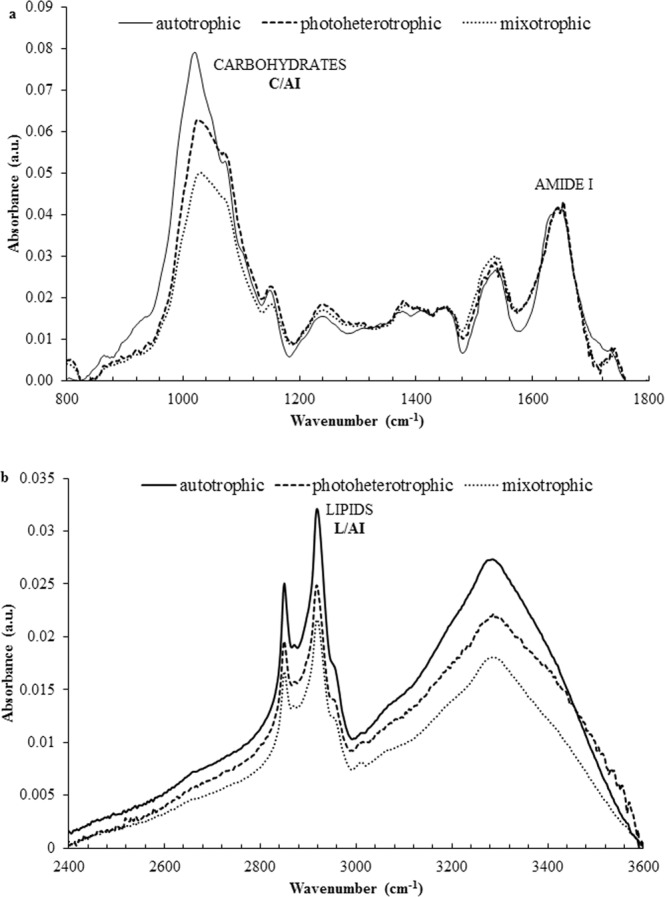
FT-IR spectra of *Tetradesmus obliquus* cell biomass growing under autotrophic, photoheterotrophic, and mixotrophic cultivation modes in the ranges from 800 to 1800 cm^−1^ (**a**) and from 2400 to 3600 cm^−1^ (**b**) (the results are presented as the mean spectra of at least 6 measurements from three biological replicates, the FT-IR spectra are normalized to amide I).

The results of the FT-IR spectra in the range from 800 to 1800 cm^−1^ and from 2400 to 3600 cm^−1^ are shown in Fig. 2a,b. Table 2 shows the location of the maximum of absorption bands of all *T. obliquus* cells. In the analysed range of 600–4000 cm^−1^, there are typical absorption bands characteristic for three main macromolecules: proteins, lipids, and carbohydrates. There is a carbohydrate band located in the range of 900–1200 cm^−1^, lipid bands with maximum absorption at ca. 1735 cm^−1^ and 2917 cm^−1^, and amide I and amide II bands in a protein region at ca. 1640 cm^−1^ and 1540 cm^−1^.

The spectra (Fig. 2a), show a band in the wavenumber range of 900–1200 cm^−1^ with a maximum at ca. 1025 cm^−1^ assigned to stretching vibrations of C-O-C functional groups typical of carbohydrates and at a maximum of ca. 1150 cm^−1^ assigned to stretching vibrations of C-O-C functional groups. The band appearing in the spectra with a maximum at ca. 1240 cm^−1^ was assigned to asymmetrical stretching vibrations of the P=O bond located in the phosphodiester groups of nucleic acids and phospholipids.

The cellular biomass spectra of both species of green algae comprise two bands located between 1500–1700 cm^−1^ with a maximum at ca. 1640 cm^−1^ and 1540 cm^−1^, referred to amide I and amide II bands, respectively, and corresponding to symmetrical stretching vibrations of C=O functional groups, symmetrical bending vibrations of N-H groups, and symmetrical stretching vibrations of C-N groups. The amide I and amide II bands are regions characteristic for proteins. Additionally, in the range of 1500–1700 cm^−1^, there are other bands corresponding to vibrations that can be assigned to protein functional groups with a maximum at ca. 1455 cm^−1^ and at 1380 cm^−1^, corresponding respectively to asymmetric bending vibrations of methyl and methylene groups and symmetrical bending vibrations of methyl and methylene groups and carboxylic groups. Additionally, there is a band in the range of 800–1800 cm^−1^ with a maximum at ca. 1735 cm^−1^, which is characteristic for symmetrical stretching vibrations of ester groups (C=O) in fats and fatty acids.

Figure 2b presents FT-IR spectra of *T. obliquus* cellular biomass in the range of 2400–3600 cm^−1^ with a band corresponding to asymmetrical stretching vibrations of C-H groups with a maximum at ca. 2920 cm^−1^. All spectra of the cellular biomass of the analysed green algae contain a broad band at 3000–3600 cm^−1^ with a maximum at ca. 3290 cm^−1^ typical for stretching vibrations of O-H groups derived from water and stretching vibrations of N-H functional groups of proteins. Table 2 presents the detailed location of the maximum of absorption bands (cm^−1^) characterizing lipid, carbohydrate, and protein regions in all the *Tetradesmus obliquus* culture variants.

**Figure 3:**
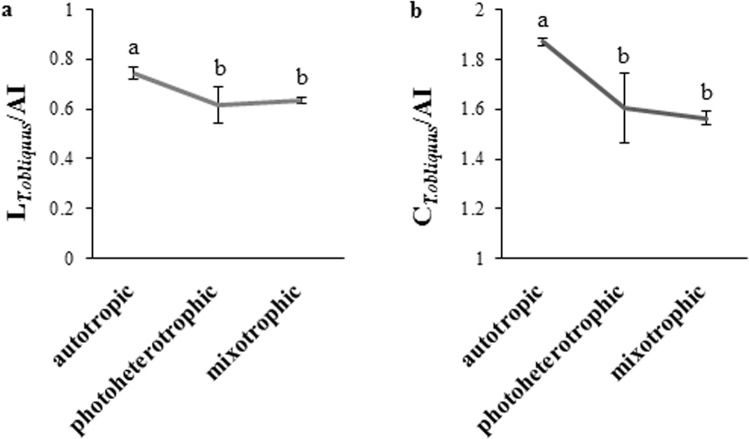
Comparison of the ratio of the intensity of the lipid absorption bands with a maximum at 2920 cm^−1^ (L_*T.obliquus*_) to the amide I band at maximum at 1640 cm^−1^ (AI) assigned as (L_*T*__.__*obliquus*_/AI) (**a**) and the ratio of the intensity of the absorption band of carbohydrate with a maximum at 1020 cm^−1^ (C_*T*__.__*obliquus*_) to the amide I band at maximum at 1640 cm^−1^ (AI) assigned as (C_*T*__.__*obliquus*_/AI) (**b**) of *T. obliquus* at different nutritional strategies (the results are presented as the mean of at least 6 measurements from three biological replicates, error bars represent standard deviation, means followed by the same letter are not significantly different; Tukey HSD test, α = 0.05).

The effect of the cultivation modes on the lipid and carbohydrate content in *T. obliquus* biomass can be observed from the ratio of the intensity of the lipid absorption bands with a maximum at 2920 cm^−1^ to the amide I band (1640 cm^−1^) for *T. obliquus* which were assigned as L_*T*__.__*obliquus*_/AI. In turn, the ratio of the intensity of the absorption band of sugars with a maximum at 1020 cm^−1^ to the amide I band (1640 cm^−1^) for *T. obliquus* was assigned as C_*T*__.__*obliquus*_/AI (Fig. 3a,b). Based on the L_*T*__.__*obliquus*_/AI ratio with a value of 0.74, the highest lipid content was found in *T. obliquus* cell biomass in the autotrophic culture. Based on the values of the ratio L_*T*__.__*obliquus*_/AI of 0.61, the lowest lipid content was detected in *T. obliquus* from the photoheterotrophic culture. The *T. obliquus* biomass from the autotrophic culture was characterised by the highest carbohydrate content, which was indicated by the C_*T*__.__*obliquus*_/AI ratio of 1.86.

### Analysis of lipid, protein, and carbohydrate content

The biochemical composition has been determined with standard Bligh and Dyer^18^, Trevelyan^19^, and Kjeldahl^10^ methods. The cell composition of *T. obliquus*, productivities, and daily productivities of individual cell components are shown in Table 1.

The results showed that there were no statistically significant differences in the lipid content in the *T. obliquus* cells. However, the highest lipid productivity (568.7 mg L^−1^) was obtained for the mixotrophic cultures. It was observed that the nutritional strategy had a significant effect on the protein content in the *T. obliquus* cells (Table 1). The highest protein content (45.15%) was obtained for the mixotrophic culture conditions, which resulted in high protein productivity. The mixotrophic conditions contributed to a significant decrease in the carbohydrate content, compared to the autotrophic mode. In all variants of the experiment, the high carbohydrate productivity ranged from 896.6 mg·L^−1^ to 1259.6 mg·L^−1^ for the photoheterotrophic and autotrophic conditions, respectively. The daily protein and lipid productivities (shown in Table 1) increased in the following order: autotrophic<photoheterotrophic<mixotrophic. In contrast, the daily carbohydrate productivity increased in the following series: mixotrophic<photoheterotrophic <autotrophic.

### Analysis of fatty acid profile Table 1

 presents general characteristics of the fatty acid profile in *T. obliquus* cells as well as specification of the percentage of C16-C18 acids and the sum of saturated (SFA), monounsaturated (MUFA), and polyunsaturated (PUFA) fatty acids among the C16-C18 acids in all the culture variants.

In the control cultures, photoheterotrophic cultures, and mixotrophic cultures, C16-C18 fatty acids account for almost 80% of all fatty acids detected in *T. obliquus* cells. Depending on the culture conditions, the content of saturated, monounsaturated, and polyunsaturated fatty acids C16-C18 varies as well. The highest SFA and MUFA percentage was found in cells from the autotrophic culture. The differences between the SFA and MUFA percentages in all the culture variants were statistically significant. In the photoheterotrophic and mixotrophic cultures, there was a significant increase in the percentage of polyunsaturated fatty acids (PUFA) relative to the autotrophic culture.

**Figure 4:**
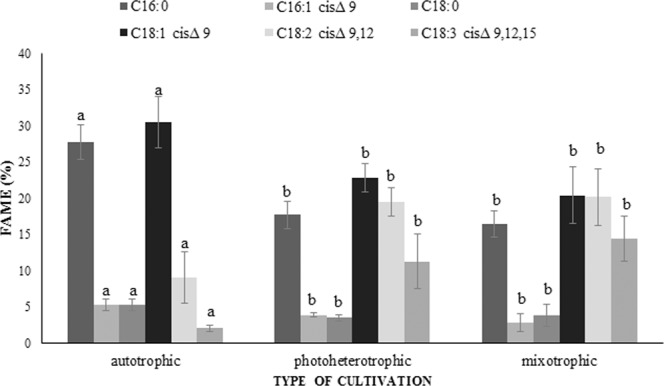
Influence of the nutritional strategies on the fatty acid profile in *Tetradesmus obliquus* cells (the results are presented as the mean of at least 6 measurements from three biological replicates, error bars represent SD, means followed by the same letter are not significantly different; Tukey HSD test, α = 0.05).

Depending on the *T. obliquus* culture conditions, the abundance of individual fatty acids varied. The effect of the culture conditions on the percentage of C16-C18 fatty acids in the cellular biomass is presented in Fig. 4. The percentage of C16:0 and C16:1, cisΔ_9_ differs significantly between the culture variants. The highest percentage of C16:0 was detected in cells from the autotrophic culture (27.77%). The photoheterotrophic culture and mixotrophic culture were characterised by a significant decrease in the percentage of C16:0, compared with the control culture.

The *T. obliquus* cells exhibited the lowest percentage of C16:1, cisΔ_9_, i.e. 5.28%, 3.91%, and 2.83% in the autotrophic, photoheterotrophic, and mixotrophic cultures, respectively. A significant decrease in the percentage of C16:1, cisΔ_9_ was noted in the photoheterotrophic and mixotrophic cultures, compared with the control. There were no statistically significant differences between the percentage of C16:0 and C16:1, cisΔ_9_ in cells from the photoheterotrophic and mixotrophic cultures.

The highest percentage of C18:0 was detected in *T. obliquus* cells from the control culture (5.30%). There was also a significant decline in the percentage of C18:0 in cells from the other experimental cultures. The lowest C18:0 abundance, i.e. 3.52% was detected in cells from the photoheterotrophic cultures. There were no significant differences in C18:0 percentage between cells from the photoheterotropic culture and the mixotrophic culture.

The highest percentage of C18:1, cisΔ_9_ (30.48%) was detected in *T. obliquus* cells growing in the autotrophic culture. In turn, a significant decrease in the percentage of C18:1, cisΔ_9_ was noted in the others experimental variants. In both cases, the molasses supplementation contributed to an increase in the content of polyunsaturated fatty acids. There were no significant differences in the percentage of C18:2, cisΔ_9,12_ ω6, and C18:3, cisΔ_9,12,15_ ω3 between cells from the photoheterotrophic and mixotrophic cultures. The highest amount of C18:2, cisΔ_9,12_ ω6 (20.19%) and C18:3, cisΔ_9,12,15_ ω3 (14.37%) was detected in the mixotrophic culture. Additionally, *T. obliquus* cells growing in the mixotrophic culture conditions were characterised by a significantly higher level of C18:2, cisΔ_9,12_ ω6 relative to that in the control. In turn, the ratio of the ω6:ω3 acids in *Tetradesmus obliquus* was 4:1, 1.6:1 and 1.5:1 in the autotrophic, photoheterotrophic, and mixotrophic cultures, respectively (Table 1).

## Discussion

The short adaptation phase in the photoheterotrophic and mixotrophic culture conditions may result from the presence of organic carbon compounds derived from beet molasses in the culture medium. The short lag phase was caused by the rapid transport and diffusion of organic carbon in the form of reducing sugars into the cell using membrane transport proteins^20^. The most assimilable hexoses, which are present in beet molasses, are rapidly and directly utilized by cells and converted into the central carbon metabolism, providing a large amount of energy in the form of ATP^11,20,21^. Zhang *et al*.^22^ also observed that, especially at a low concentration in culture medium, glucose shortened the lag phase, as it was assimilated easily during the cultivation.

In comparison to the autotrophic mode, an increase the final biomass concentration and specific growth rate was observed in the photoheterotrophic and mixotrophic culture conditions. In the present studies, the mixotrophic *T. obliquus* culture conditions were characterized by the highest biomass yield and specific growth rate as well as the shortest biomass doubling time. As reported by Yang *et al*.^23^, simultaneous supply of light, CO_2_, and an organic carbon source to the medium induced the mixotrophic growth of algae and resulted in the highest final biomass yield. It can be assumed that the highest biomass yield was the result of simultaneous photosynthetic carbon and organic carbon uptake. Organic carbon is supplied to algal cells through active transport and phosphorylation^24^. Kamalanathan *et al*.^25^ reported that the photosynthetic activity of *Scenedesmus obliquus* during molasses-based mixotrophic growth was comparable with that of cells in photoautotrophic cultures. The researchers showed that *Scenedesmus obliquus* cultured in mixotrophic conditions had a fully functional photosynthetic apparatus.

In *T. obliquus* cells cultured in autotrophic conditions, the highest carbohydrate content was detected using both spectrophotometric analysis and infrared spectroscopy. Cells cultivated in autotrophic growth conditions are mainly a source of carbohydrates^26^. Thus, the presence of a sufficient amount of CO_2_ significantly influences the carbohydrate accumulation in microalgae. Additionally, the increasing CO_2_ concentration has an impact on carbohydrate accumulation^12,27^.

The *Tetradesmus obliquus* cells had high-protein content in the photoheterotrophic and especially in the mixotrophic growth conditions. This result confirms the fact that both the nitrogen and carbon sources present in the medium target the metabolism towards protein synthesis and intensification of cell growth^28^. Beet molasses is mainly a source of organic compounds in the form of sucrose and reducing sugars. Besides organic carbon, molasses contains nitrogen in the form of nitrates, nitrites, and amino acids (Table 3). Additional nitrogen originating from the source is directly involved in the synthesis and accumulation of cellular proteins^10^. Protein is the main component constituting up to 60% of marine and freshwater algal cells and cyanobacterial cultures growing in nitrogen conditions^29^. Nitrogen is responsible for stimulation of the growth of algae and ensures the synthesis of proteins involved in carbon assimilation^30^. At N-repletion in the culture medium, *Chlamydomonas reinhardtii* cells were characterized by an increase in transcripts for genes encoding photosynthetic complexes, electron transport chain, photosynthetic antenna systems, pigments synthesis, and Calvin-Benson cycle^31^. N-repletion promotes RuBisCO synthesis, which is the largest reservoir of nitrogen in cells in stress conditions^32,33^. In nitrogen availability conditions, algal cells were also characterized by high content of free intracellular nitrogen compounds, i.e. free nitrates and free amino acids^34^. The control of metabolism and targeting the metabolic pathways towards synthesis and accumulation thereof can be an opportunity for development of many branches of industry, e.g. pharmacy, medicine, and cosmetics.

The presence of an organic form of carbon in the culture medium, most often glucose^9^, has an impact on the lipid content in algal cells. Additionally, nitrogen deficiency in the environment causes redirection of the metabolism towards the synthesis of reserve material in the cell in the form of triacylglycerols or starch^35^. Generally, lipid synthesis in cells occurs upon depletion of nitrogen in the environment^36,37^, at a high carbon/nitrogen ratio in the culture medium (C/N)^38^. The results obtained by modified Bligh & Dyer method indicate that *T. obliquus* cells have similar lipid content in all culture variants. In contrast, the qualitative FTIR analysis of the cells showed reduced lipid content in *T. obliquus* cells from the photoheterotrophic and mixotrophic cultures, compared to the autotrophic cultures. The differences in the results of the cell lipid content are a consequence of the method used for the analysis. The Bligh & Dyer extraction method is used for all extractable compounds in solvents, including chlorophyll^39^, and the FTIR determination of the lipid content was based on the stretching vibration band of the methylene group (CH_2_) characteristic for lipids^40^. This study indicates that the photoheterotrophic and mixotrophic culture of *T. obliquus* provided the cells with optimal environmental conditions and all necessary biogenic elements, mainly carbon and nitrogen without inducing stress, which results in reduced lipid content. The lipid content in the *T. obliquus* cells are close to the average lipid content in algal cells growing in optimal environmental conditions, i.e. 25.5% suggested by Hu *et al*.^41^ and 23% (green algae) proposed by Griffiths & Harrison^42^. Accumulation of lipids is a protective mechanism in algal cells induced by exposure to stress. In optimal environmental conditions, algal cells mainly contain polar membrane lipids^35^. The present results agree with those reported by Yan *et al*.^36^, who demonstrated that the presence of nitrogen in molasses did not affect lipid accumulation in *Chlorella protothecoides* cells in heterotrophic growth conditions, and lipid accumulation was observed only in the case of a glucose-enriched medium with reduced nitrogen content.

Regardless of the culture conditions, the fatty acid profile in *T. obliquus* is dominated by acids containing from 16 to 18 carbon atoms in the carbon chain. This is typical for Chlorophyta, which is confirmed in the literature^43^. In the photoheterotrophic and mixotrophic modes, the percentage of individual fatty acids and the content of SFA, MUFA, and PUFA groups were different from those in the autotrophic culture conditions. Literature data on the fatty acid profile in Chlorophyta under beet molasses supplementation are very scarce. Only two studies describe the effect of molasses of unknown origin on the fatty acid profile in *Chlorella protothecoides*^36^ and *Chlorella minutissima*^44^. Yan *et al*.^36^ observed that molasses addition to culture medium caused an increase in the linoleic acid content. The fatty acid content is variable; every species described in the literature functions differently in different environmental conditions. The fatty acid composition in *T. obliquus* was affected by additional nitrogen in the culture medium. Nitrogen deficiency in the culture medium can decrease the content of linolenic acid in algal cells. This is directly linked to the activity of the gene encoding the - Δ15 desaturase enzyme involved in formation of linolenic acid^45^. The activity of enzymes involved in the synthesis and transformation of fatty acids depends on the presence of nitrogen in the culture medium. *T. obliquus* cells growing under N supply are characterized by high content of linolenic acid, suggesting increased Δ15 desaturase activity. *T. obliquus* cells growing in the presence of beet molasses (both photoheterotrophic and mixotrophic culture conditions) produced high amounts of oleic acid, linoleic acid C18: 2, cis_Δ 9,12,_ω6 (LA) and α-linolenic acid 18: 3, cis_Δ 9,12,15_, ω3 (ALA). Linoleic acid and α-linolenic acid are essential fatty acids (EFA). LA is a precursor for the synthesis of ω6 long chain acids, including arachidonic acid (ARA) and ALA is converted to long chain ω3 acids such as eicosapentaenoic acid (EPA) and docosahexaenoic acid (DHA)^14^. Essential fatty acids from both the ω3 and ω6 families are indispensable for the proper function of the human organism and must be provided through dietary intake, as they cannot be synthesized in the human body^14–16^. The major dietary sources of LA are vegetable oils sunflower, corn, safflower, peanut, and olive oil, whereas ALA it is found, in vegetable oils, seeds, nuts, legumes, grains, fruits, and some wild plants, in addition to algae. ALA is the most important ω3 fatty acid in the human diet. It takes part in reduction of the risk of degenerative diseases e.g. heart and cancer diseases, arthritis, skin conditions, diabetic neuropathy, immune function, and premenstrual syndrome^46^.

Omega 6 acids are supplied to the human organism in substantial amounts, whereas the amount of ω3 acids is very low. This leads to a disturbed ratio of ω6:ω3 fatty acids. Inappropriate proportions may contribute to the development of many serious diseases. The World Health Organization (WHO) recommends consuming products in a diet with a ω6: ω3 acid ratio lower than 10:1^47,48^. The optimal ratios fluctuate between 4:1 and 1:1^49^. *Tetradesmus obliquus* is characterized by having ratios of 4:1, 1.6:1 and 1.5:1 of ω6:3 in the autotrophic, photoheterotrophic, and mixotrophic cultures, respectively. Therefore, *T. obliquus* cells derived from molasses-supplemented cultures seem to be suitable for dietary supplementation of essential fatty acids.

With algal capability of simultaneous biosynthesis of several metabolites, algal cell biomass is used in many fields of biotechnology. Algal species exhibit this ablilty include e.g. *Phaeodactylum tricornutum*, which are able to synthesize lipids and fucoxanthins simultaneously^50^ and *Odontella aurita*, which synthesize EPA acid and fucoxanthin^51^. Due to the high protein content and productivity as well as the presence of essentials fatty acids with a favourable ω6:ω3 ratio, *Tetradesmus obliquus* cells growing on a beet molasses-supplemented medium, are important for biotechnological applications and can be used in human and animal nutrition and supplementation.

## Materials and Methods

### Characterization of the species and algal culture conditions

The green algae *Tetradesmus obliquus* (strain No. 276-1) were obtained from the Culture Collection of Algae (SAG) at Göettingen University. The inoculation culture of *T. obliquus* was carried out in Erlenmeyer flasks containing 50 cm^3^ and successive 250 cm^3^ of liquid mineral Bold’s Basal Medium (BBM). During the pre-cultivation ambient temperature (25 °C), light intensity (80 μmol photons m^−2^ s^−1^), photosynthetic radiation (fluorescence lamps - Philips  Aquarelle) and continuous light (24 h light/0 h dark) were monitored. The *T. obliquus* cells were cultured on a laboratory shaker to prevent sedimentation.

Batch cultures of *T. obliquus* were established in BIOSTAT PBR 2 S Sartorius Stedim Biotech photobioreactors by application of the inoculum obtained in the preliminary culture. The initial optical density (OD_650_) was 0.5. The *T. obliquus* cells were cultured in autotrophic, photoheterotrophic, and mixotrophic growth conditions, which corresponded to the aerated culture (control), molasses-supplemented culture, and aerated molasses-supplemented culture. Each culture variant was performed in three independent biological replicates. Filtered sterile atmospheric air at a flow of 12 L h^−1^ was constantly supplied to the aerated cultures (autotrophic and mixotrophic cultivation modes). Molasses at a concentration of 10 g L^−1^ was used as a substrate for enrichment of the photoheterotrophic and mixotrophic cultures. The beet molasses was provided by a local sugar factory. Its composition is presented in the Table 3. The batch cultures were carried out at a temperature of 25 ± 1 °C under continuous light with light intensity of 80 µmol photons m^−2^ s^−1^ for 12 days. The biomass was harvested by centrifuging after 12 and used for further biochemical analyses.

### Measurement of cell biomass concentration and determination of growth curves

During the cultivation in the photobioreactors, spectrophotometric measurements of optical density (OD_650_) were carried out every 24 h using a Cary 300/Biomelt spectrophotometer. Based on the calibration curve equation for *T. obliquus* (DCW = 303.21 × OD_650_ − 2.354, *R*^2^ = 0.9958) and the determined optical density values, the theoretical dry cell weight (expressed in g L^−1^) was determined during the consecutive days of cultivation, demonstrating the growth of the cultured *T. obliquus* cells^52^.

**Table 3:** Characteristics of beet molasses.

Parameters	Value
Apparent dry matter, °Bx	80.1
Apparent sucrose, %	49.4
pH	8.6
Reducing sugars, %	0.16
Total nitrogen, %	1.98
SO_2_, %	0.009
Ca and Mg (salts), %	0.14
Volatile acids, %	0.94
Total organic carbon, %	34.6
Total nitrite, mg/kg	<0.667
Total nitrate, mg/kg	2600
Glutamic acid, g/kg	29.6
Aspartic acid, g/kg	3.02

### Determination of the basic growth parameters

The basic parameters for evaluation of unicellular algal growth, i.e. biomass yield (the final biomass concentration), the specific growth rate, and the biomass doubling time, were used to assess the cell growth in the culture. These parameters are used to describe the kinetics of unicellular algal growth in batch cultures.

The specific growth rate (*µ*) is a measure of the rate of algal growth in the logarithmic phase. It was calculated using Eq. 1 for the first three days of cultivation.

1 $$\mu ({{\rm{day}}}^{-1})=\,\mathrm{ln}({{\rm{N}}}_{2}/{{\rm{N}}}_{1})/({{\rm{T}}}_{2}-{{\rm{T}}}_{1})$$

where: N_1_ and N_2_ are the optical density values at OD_650_ and time T_2_ and T_1_, respectively.

The biomass doubling time (*T*_*d*_) was calculated based on the specific growth rate using Eq. 2 during the first 72 hours of cultivation. The biomass doubling time was expressed in hours.

2 $${T}_{d}=(\mathrm{ln}\,2/\mu )\times 24$$

where: *µ* is specific growth rate.

The biomass yield after the cultivation was expressed in g L^−1^. In turn, the daily biomass productivity was expressed in g L^−1^ day^−1^^10,53,54^.

### Analysis of biochemical composition – lipids, proteins, and carbohydrates

Lipid content was determined in the algal biomass with the gravimetric method, which is a modification of the Bligh and Dyer method^18,52^. An ultrasound-assisted lipid extraction method with the use of a chloroform and methanol mixture in a volume ratio of 1:2 v/v was employed. After the extraction, the solvent was evaporated on a Heidolph vacuum evaporator at a temperature of 40 °C. The lipid content was determined gravimetrically and expressed in % (m/m); next, the extract was suspended in 99% n-hexane (HPLC grade, Sigma-Aldrich). The samples were stored in a freezer until further determinations.

The total content of simple sugars was determined colorimetrically with the anthrone method^19^. A 2% anthrone solution in 98% H_2_SO_4_ was added to the cell suspension. The samples were heated in a boiling-water bath for 10 min. After cooling to room temperature, the absorbance of the samples at 620 nm was measured spectrophotometrically with using Cary 300/Biomelt. The content of soluble simple sugars (mg ml^−1^) was determined using the calibration curve prepared for the glucose standard. The carbohydrate content was expressed in % (m/m).

The Kjeldahl method was used to determine the total nitrogen and protein content in the algal cells^10^. To calculate the total protein content, the total nitrogen content was multiplied by an algae-specific nitrogen-to-protein conversion factor of 5.95^55^ in accordance with Eq. 3.

3 $${\rm{Total}}\,{\rm{protein}}[ \% \,{\rm{d}}.{\rm{w}}.]={\rm{N}}\times 5.95$$

where: N - total nitrogen content [% d.w.].

Protein, lipid, and carbohydrate productivities (mg L^−1^), the daily protein, lipid, and carbohydrate productivities (mg L^−1^ day ^−1^) were calculated based on the percentage content of cellular components and the biomass yield (g L^−1^).

### Determination of fatty acid methyl ester content

The mixture of fatty acid methyl esters was obtained by transesterification of lipids with methanol in the presence of catalysts^13^. Briefly, crude oil suspended in n-hexane (HPLC grade, Sigma-Aldrich) was evaporated and 0.5 M KOH-methanol (Sigma-Aldrich) was added and hydrolyzed at the temperature of 80 °C. Esterification was carried out by adding 10% BF_3_ in methanol (for GC derivatization, Fluka). The reaction was performed at 100 °C for 20 min. Next, 99% n-hexane (HPLC grade, Sigma-Aldrich) and a saturated NaCl solution (Sigma-Aldrich, Supelco) were successively added. Separation of methyl esters and determination of fatty acids was performed on a Trace GC Ultra gas chromatograph coupled with ion trap mass spectrometer ITQ 1100 (GC/MS) from Thermo Scientific using a Rtx-2330 column, with a length of 105 m, inner diameter of 0.25 mm, and film thickness of 0.25 μm. Helium at a flow rate of 2.4 ml/min was the carrier gas. To determine the fatty acid composition, separation of the mixture of standard solutions − 37 Component FAME Mix solutions (Sigma-Aldrich, Supelco) was carried out.

### Qualitative analysis of algal biomass with the use of FT-IR spectroscopy

The infrared spectrometric analyses were carried out using an FT-IR spectrometer (Fourier Transform Infrared) - Nicolet 6700 (Thermo Electron Corporation, USA) with a single-reflective ATR (Attenuated Total Reflection) diamond-attachment. The spectrometer facilitates measurement in the infrared range from 4000 to 600 cm^−1^ with a resolution of 4 cm^−1^. The number of scans was 128. The measurements demonstrated three spectra for each culture, which was an independent biological replication. In total, 9 spectra were obtained for each cultivation variant. The spectra were normalized to the amide I band at 1640 cm^−1^,which is the most intense band and, on their basis, the mean representative FT-IR spectrum was determined for each variant of the culture^40^. The content of carbohydrates and lipids in the biomass was assessed by determination of the intensity of the sugar band (1025 cm^−1^) to the protein band (amide I band − 1640 cm^−1^) and the ratio of the intensity of the lipid band (2917 cm^−1^) to the protein band (amide I band − 1640 cm^−1^).

### Statistical analysis of results

Statistical analysis of the investigation results was carried out in the STATISTICA 12 program (StatSoft Inc., USA). To determine the impact of the nutrition strategy on the analysed quantitative and qualitative parameters of algal biomass, two-factor analysis of variance was carried out, and the significance of the differences was tested at a significance level of p ≤ 0.05 using the post-hoc Tukey test.
